# Comparing the efficacy of two antibiotic cocktails in decontamination of cardiovascular tissues

**DOI:** 10.1007/s10561-025-10198-9

**Published:** 2025-10-30

**Authors:** Alina Levy, Helit Cohen, Nadezda Savieva, Meytal Neeman-Azulay, Natasha Belausov, Ehud Raanani, David Mishali, Rachel Kornhaber, Michelle Cleary, Jonathan Esensten, Sharon Amit, Ayelet Di Segni

**Affiliations:** 1 Present Address: Sheba Tissue and Cell Bank, Sheba Medical Center, Tel Hashomer, 52621 Ramat Gan, Israel; 2 Clinical Microbiology Laboratory, Sheba Medical Center, Tel Hashomer, 52621 Ramat Gan, Israel; 3 Advanced Biotherapy Center, Sheba Medical Center, Tel Hashomer, 52621 Ramat Gan, Israel; 4 Cardiology Division, Sheba Medical Center, Tel Hashomer, 52621 Ramat Gan, Israel; 5 School of Nursing, Paramedicine and Healthcare Sciences, Charles Sturt University, Bathurst, NSW Australia; 6 School of Health, University of New England, Armidale, NSW Australia

**Keywords:** Cardiovascular, Allografts, Decontamination, Antibiotic, Tissue bank

## Abstract

Cardiovascular allografts are essential for patients with severe cardiovascular diseases. Yet, microbial contamination of the grafts poses a life-threatening risk to recipients. Tissue banks utilize various decontamination methods during cardiovascular tissue processing, often involving antibiotic solutions. This study compares the efficacy of an in-house prepared antibiotic cocktail (tissue bank cocktail) and a commercially available solution (BASE.128) in decontaminating cardiovascular tissues. For this study, the efficacy of the two antibiotic cocktails was compared through quantitative comparisons against challenge microorganisms, and retrospective analysis of routine sterility tests. Both solutions demonstrated comparable decontamination efficiency against challenge strains, achieving significant reductions in bacterial load. However, retrospective sterility tests revealed that while both antibiotic solutions were highly effective in decontaminating cardiovascular allografts, the use of BASE.128 followed a tenfold increase in contamination rates compared to the tissue bank cocktail, primarily due to a slow-growing non-tuberculous mycobacteria strain. These findings highlight the importance of tailored decontamination protocols that consider prevalent microbial contaminants while preserving tissue quality.

## Introduction

Heart valve allografts are integral to modern medicine, providing a lifesaving solution for patients experiencing severe cardiovascular diseases. Tissue banks (TBs) collect, process, and store donated human heart valves and cardiovascular (CVS) tissues, ensuring a readily available supply for patients in need (Castells-Sala et al. 2024; Serafini et al. 2016). The risk of microbial contamination in CVS transplants, can pose significant threats to recipients (CDC 1997; Kuehnert et al. 1998; Witten et al. 2023). As a result, microbiological contaminations found during the processing of cardiovascular tissues have led to substantial losses, with as much as 25% of heart valves being discarded in 2019 by European banks due to this contamination (Zahra et al. 2019).

To mitigate these risks, tissue banks worldwide employ various decontamination methods, typically involving incubation in antibiotic solutions (Germain et al. 2016; Suss et al. 2018). However, in the absence of standardized guidelines, the composition and concentrations of antibiotic cocktails, along with treatment duration and temperature, vary across different tissue banks. The combination of these factors not only determines the efficacy of decontamination (de By et al. 2012; Germain et al. 2016, 2010; Serafini et al. 2016; Suss et al. 2018; Zahra et al. 2019), but can also influence cell viability and the biological structure of the tissue (Gall et al. 1998; Heacox et al. 1988). Furthermore, residual levels of antibiotics have been detected in heart valves despite thorough rinsing, cryopreservation and thawing (Buzzi et al. 2014; Jashari et al. 2011). Although these residual levels are low and unlikely to negatively impact graft recipients, their carryover to enrichment media used in sterility tests may result in false-negative results (Buzzi et al. 2014). Given these complexities in decontamination procedures and their potential implications for both tissue quality and sterility testing, decontamination protocols need to be tailored to address the most common microbial contaminants identified in each tissue bank, while preserving tissue quality (de By et al. 2012).

The Sheba Tissue and Cell  Bank, established in 2018, has to date procured and processed 191 hearts, generating 369 CVS tissues (valves and bifurcations). Until the end of 2023, the CVS decontamination process utilized an in-house tissue bank-prepared antibiotic cocktail solution (TB cocktail), consisting of six antimicrobial agents targeting a broad spectrum of microorganisms. However, considering the potential adverse effects of antibiotics on CVS tissue integrity (Gall et al. 1998; Heacox et al. 1988), we transitioned to BASE.128, a commercially available, ready-to-use decontamination solution, containing fewer antibiotics at lower concentrations.

In this study, we compare the effectiveness of the in-house TB cocktail and the commercial BASE.128 solutions using two approaches: (1) a quantitative comparison of decontamination efficiency against representative challenge microorganisms, and (2) a retrospective analysis of routine sterility tests performed prior to CVS final packaging and cryopreservation.

## Material and methods

### Ethics statement

This study was conducted in compliance with all relevant ethical guidelines concerning the use of human tissues (SMC-1011-24). The Sheba Tissue and Cell Bank is a public tissue bank that operates under stringent ethical and legal frameworks governing tissue collection, storage, and distribution, in accordance with the Israeli Ministry of Health regulations (Ministry of Health Pharmaceutical Division 2023). All tissue samples were anonymized to ensure donor privacy and confidentiality. The various stages of the process are regulated by the Ministry of Justice of Israel and the Ministry of Health (Frenkel 2008).

### Tissues

During the study period, 369 CVS tissues were dissected and processed in the Sheba Tissue and Cell Bank, with 296 tissues decontaminated using the TB cocktail, and 73 tissues were decontaminated using BASE.128, corresponding to 155 and 36 hearts procured, respectively.

### Decontamination

Each CVS tissue was incubated in 500 ml of decontamination solution at 4 °C for 48 h. Following decontamination, the tissue was rinsed by incubation in Medium 199 for 30 min at room temperature. The final product was aseptically packed in clean rooms under a class A environment, and then cryopreserved.

The TB antibiotic cocktail was used from January 2018 to November 2023. The cocktail was prepared as follows: Medium 199 (Gibco): 8.8% (v/v); 1 M Sodium Bicarbonate: 4% (v/v); Vancomycin: 500 mg/L; Ciprofloxacin: 200 mg/L; Gentamicin: 80 mg/L; Cefuroxime: 250 mg/L; Colistin: 1,000,000 units/L; and Amphotericin B: 100 mg/L (added just before use) in sterile water. BASE.128 (Alchimia Srl, Italy) has been used since December 2023 to April 2025. A comparison of the antibiotic composition in the two solutions is presented in Table 1.

**Table 1:** Antibiotic concentrations in the tissue bank cocktail versus BASE.128

Antibiotic	TB cocktail	BASE.128 (Alchimia)
Vancomycin	500 mg/L	128 mg/L
Gentamicin	80 mg/L	128 mg/L
Ciprofloxacin	200 mg/L	Absent
Cephalosporin	Cefuroxime, 250 mg/L	Cefotaxime, 128 mg/L
Colistin	1,000,000 units/L	Absent
Amphotericin B	100 mg/L	20 mg/L
Base Medium	Medium 199	RPMI 1640

*TB* tissue bank

### Quality control—sterility test

During the dissection process, five tissue fragments measuring approximately 0.5 cm^2^ in surface area were cut from each tissue sample. These fragments underwent the complete production process with the tissue. At the end of the process, one fragment was minced and transferred to BacT/ALERT PF Plus (bioMerieux, France, cat# 410,853) and FN Plus (cat#410,852) bottles, which were subsequently incubated in the Bact/Alert Virtuo system (bioMerieux, France) as described below. An additional fragment was tested for the presence of mycobacteria by standard mycobacterial culture.

### Method validation—sterility test

A validation assay was performed to address the concern of antibiotic residues interfering with sterility tests, as detailed below.

### Sample preparation

Heart valve samples underwent routine dissection and decontamination procedures, after which each sample was cut into seven fragments measuring approximately 0.5 cm^2^ in surface area. Each fragment was minced for two minutes in a grinding tube containing 5 ml of saline solution using a tissue grinder (SpinAX, Axon Lab). Next, 2 ml of the minced sample was transferred to each BacT/ALERT PF Plus and FN Plus bottle, followed by microbial inoculation to detect aerobic and anaerobic microorganisms, respectively.

### Microbial inoculation

Each test bottle was exogenously inoculated with approximately 125 Colony Forming Units (CFU) of a test strain or saline, which served as a negative control. The microorganism strains tested were: *Staphylococcus aureus* (ATCC 25923), *Streptococcus agalactiae* (ATCC 27956), *Escherichia coli* (ATCC 25922), *Bacillus cereus* (from the Sheba Tissue and Cell Bank Clinical Microbiology Laboratory), *Clostridium perfringens* (ATCC 13124), *Candida albicans* (ATCC 14053). The strains were selected in accordance with pharmacopeia guidelines (USP71), which provide standards for microbial testing. To ensure clinical relevance, some strains were obtained from the American Type Culture Collection (ATCC), while others were representative of hospital flora. Notably, Clostridium was included as a validation strain only and has never been identified in our heart valve tissues. This distinction contributes to the low discard rate observed in our bank, as tissue is not automatically discarded solely based on pre-antibiotic identification of virulent organisms if subsequent post-decontamination cultures are sterile.

### Microbial detection

The bottles were incubated in the BacT/ALERT Virtuo system for 14 days, and Time-To-Detection (TTD) (the time required for automated detection of microbial growth) was documented to validate and compare the survival of the challenge bacteria in the presence of antibiotic-treated CVS samples.

### Process validation—decontamination

The efficiency of the two antibiotic solutions against selected microorganisms was tested as follows:

Spike solutions

A 10 ml saline solution was inoculated with 10^4^ CFU/ml of* B. cereu*s or 10^6^ CFU/ml of* S. aureus*. A saline solution without challenge bacteria served as the negative control.

Sample preparation and external contamination.

The decontaminated aseptic heart valve was cut into fragments measuring approximately 0.5cm^2^ in surface area. Two fragments were incubated in each spike solution or negative control for one hour at 37 °C. After one hour, the tissues were removed from the spike solutions and underwent routine decontamination with either TB cocktail or BASE.128.

Decontamination efficiency

The heart valve fragments were sampled by an eSwab (Copan, Italy, 480CE) upon removal from the spike solution (initial bioburden) and at the end of the decontamination process. 100 µl of each sample was plated onto Tryptic Soy Agar (TSA, *S. aureus*) or Blood Agar (*B. cereu*s) plates. The plates were incubated at 37 °C for 24 h, after which colonies were counted. Decontamination efficiency was determined by calculating the reduction in CFU counts at the end of the process relative to the initial bioburden.

Statistical analysis

Statistical analysis of the difference between the treatments was performed using MedCalc Software Ltd.’s Odds Ratio Calculator (MedCalc Software Ltd., 2025).

## Results

### CVS tissue discard rate in the Sheba Tissue and Cell Bank

Since its establishment in 2018, the Sheba Tissue and Cell Bank has processed 369 CVS tissues. Of these, 13 tissues (3.5%) were discarded due to microbiological contamination identified at the end of the dissection process, and 29 tissues (7.9%) were discarded for other reasons, as detailed in Table 2.

**Table 2:** CVS tissues discarded in Sheba Tissue and Cell Bank

Reason for discard	Number of tissues discarded out of 369 tissues processed	% of processed tissues
Expiration date (5 years from donation date)	7	1.9
Positive donor blood culture	17	4.6
Structural defect	1	0.3
Others	4	1.1
Positive sterility test at the end of processing	13	3.5
Total	42	11.4

### Sterility test method validation

Decontamination using antibiotic solutions may lead to interference in sterility tests due to the carryover of residual antibiotics. We validated the sterility test using six representative challenge microorganisms, recommended by the American Association of Tissue Banks (2016), that were incubated separately with antibiotic-treated CVS fragments.

As shown in Table 3, both tissue TB cocktail- and BASE. 128-treated CVS fragments enabled detection of all the contaminants at a range of 6–22 CFU/ml. TTD was comparable and within the 14-day timeframe with both decontamination solutions.

**Table 3:** TTD of challenge microorganisms incubated with tissue bank cocktail or BASE.128 treated CVS fragments

Microorganism	TTD (days:hour:minutes)			
	BacT/ALERT PF plus		BacT/ALERT FN Plus	
	TB cocktail	BASE.128	TB cocktail	BASE.128
*S. aureus*	00:15:01	00:14:01	00:15:01	00:17:01
*S. agalactiae*	00:09:47	00:09:20	00:12:06	00:13:56
*E. coli*	00:10:32	00:10:24	00:10:04	00:09:42
*B. cereus*	00:09:21	00:10:40	00:10:11	00:10:24
*C. perfringens*	Not conducted	Not conducted	00:11:07	00:21:47
*C. albicans*	00:21:55	00:21:44	04:08:00	04:04:00
Saline	Undetectable	Undetectable	Undetectable	Undetectable

*TB* tissue bank, *TTD* time-to-detection

### Decontamination process validation

After demonstrating the capability of routine sterility tests to detect common microbiological contaminations in antibiotic-treated CVS tissue, we focused on two “worst-case scenario” pathogens to compare the effectiveness of the decontamination solutions: *B.* cereus spores, which represent highly infective and environmentally stable spore-forming bacteria and *S. aureus* from the skin microbiome (American Association of Tissue Banks, 2016).

As shown in Table 4, following decontamination with either TB solution or BASE.128, the bacterial load was reduced by at least two orders of magnitude, demonstrating comparable effectiveness of the two decontamination solutions against the challenge pathogens.

**Table 4:** Decontamination process validation

Bacteria	Decontamination Solution	Number of independent repeats (*n*)	Bacterial count [CFU/ml]	
			Initial bioburden	After decontamination
*Bacillus cereus* sporulated	BASE.128	3	1–2 × 10^3^	No growth
	TB cocktail	2	2.6 × 10^3^	No growth
		1	0.5 × 10^3^	No Growth
*S. aureus*	BASE.128	2	2–4 × 0^3^	No growth
		1	3.6 × 10^3^	1 × 10^1^
	TB cocktail	3	1–4 × 10^3^	No growth

*CFU* colony forming units, *TB* tissue bank, *(n*) refers to independent repeats of the process validation protocol with the same initial bioburden

### Retrospective comparison of sterility tests

**Fig. 1:**
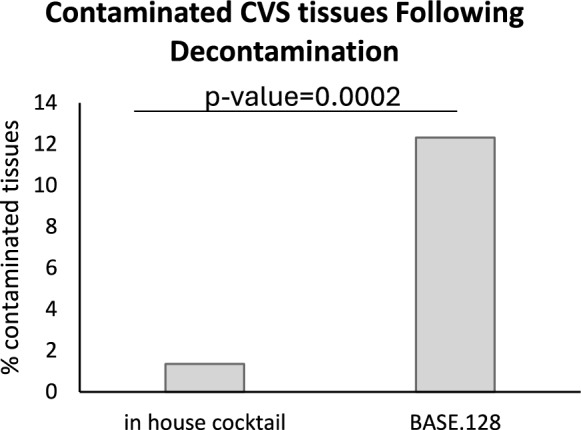
Percentage of CVS tissues found contaminated following decontamination

Sterility testing is routinely performed on each tissue processed at the Sheba Tissue and Cell  Bank prior to final packaging and cryopreservation. A retrospective comparison of the contaminations detected when using both antibiotic solutions revealed a significant tenfold increase in contamination when using BASE.128, as compared to the TB cocktail (Odds ratio 10.2305, 95% CI [3.05, 34.25], P = 0.0002) (Fig. 1).

Process validation demonstrated comparable effectiveness of the two solutions against challenge strains. However, retrospective sterility tests revealed tenfold higher contamination rates when using BASE.128 compared to TB cocktail, with slow-growing nontuberculous mycobacteria (NTM) as the most commonly detected contaminant. These observations underline the importance of continuous monitoring and careful adjustment of decontamination strategies, taking into consideration the specific microbial contaminants prevalent at each tissue bank.

Using the TB cocktail resulted in four contaminated tissues (out of 296) originating from two donor hearts. After decontamination with BASE.128, a total of 9 out of 73 tissues were found contaminated. 5 tissues dissected from 2 hearts (55% of the contaminations) were identified as *Mycobacterium simiae*. Three tissues dissected from 2 hearts (33% of the contaminations) were identified as predominantly oral colonizing bacteria. Because sampling was conducted only on the final product, it was not possible to identify the exact stage at which contamination occurred. Therefore, each graft was treated as an individual specimen rather than being grouped as tissue from a single contaminated heart. Notably, none of these bacteria were detected following decontamination with the tissue bank cocktail (Table 5). Together, the results demonstrate that both antibiotic solutions are highly effective in decontaminating CVS tissue allografts, with a potential advantage of the TB cocktail against *Mycobacterium* and possibly certain* Streptococcus* species.

**Table 5:** Bacteria identified in tissues after decontamination

TB cocktail	
Bacteria	Number of infected tissues (n = 4)
Gram-negative bacteria (no species identification)	3 (1.0%)
*Proteus mirabilis*	1 (0.3%)
BASE.128	
Bacteria	Number of infected tissues (n = 9)
*Mycobacterium simiae*	5 (6.8%)
*S.anginosus group; S. mitis/oralis; Prevotella sp.*	1 (1.4%)
*S. oralis*	2 (2.7%)
Positive anaerobic culture, no species identification	1 (1.4%)

*TB* tissue bank, % = percentage of contaminated tissues relative to the total CVS tissues processed (TB cocktail = 296; BASE.128 = 73)

## Discussion

Decontamination of heart valves within tissue banks is a critical step in ensuring the safety and viability of these allografts. To reduce the number of discarded CVS tissues and mitigate the risk of post-transplant infections, various decontamination protocols are employed during the allograft dissection process (de By et al. 2012; Germain et al. 2016). Although antibiotic treatment is the preferred decontamination method among TBs, it has several limitations (Heacox et al. 1988) and can negatively affect tissue viability. For instance, the commonly used antifungal agent Amphotericin B (de By et al. 2012) has been shown to reduce the quality of cryopreserved heart valves (Gall et al. 1998).

Rather than cellular viability itself, most experts now emphasize preservation of the extracellular matrix (ECM) and overall structural integrity as the primary determinants of graft durability (Poulis et al. 2022). Indeed, the presence of viable cells may increase antigenicity and contribute to higher immunogenic responses (Fabian et al. 2022), which has prompted some TBs to investigate decellularization strategies. At the same time, excessive loss of cellular and matrix preservation may lead to structural degeneration, which also compromises durability (Hepfer et al. 2016; Peters et al. 2024). Therefore, any decontamination solution must be carefully assessed for its impact on tissue integrity, while minimizing adverse effects on both the ECM and any residual viable cells. Finally, antibiotic-based decontamination may interfere with sterility testing performed before final packaging (Buzzi et al. 2014).

In the seven years since the Sheba Tissue and Cell Bank  began operations, 205 CVS grafts have been distributed for transplantation, with no reported cases of post-operative infection. This achievement in part corresponds to the effectiveness of the TB’s rigorous protocols, which combine thorough decontamination and sterility testing to ensure that no contaminated tissues are used in clinical procedures. Notably, discard rates due to bacterial contamination were exceptionally low, at only 1.4%, when decontamination was performed using the TB cocktail.

Although the TB cocktail demonstrated robust effectiveness, we transitioned to BASE.128 to reduce the number and concentrations of antibiotics used in the decontamination protocol, while standardizing the process with a commercially available solution. The most common contaminants in our TB are Gram-positive skin commensal bacteria, mainly *Staphylococcus* sp. BASE.128 is a ready-to-use solution that contains a broad range of antibiotics effective against these Gram-positive bacteria, making it well-suited for our needs. Additionally, BASE.128 includes amphotericin B to target fungi, at a reduced concentration compared to our previous TB cocktail.

Despite the reduced number of antimicrobial agents and lower concentrations of most antibiotics (except for Gentamicin) in BASE.128, both the TB cocktail and BASE.128 solutions demonstrated equivalent antimicrobial efficacy against *S. aureus* and *Bacillus cereus*. Both formulations achieved a minimum 2-log10 reduction in bacterial load, meeting the American Association of Tissue Banks criteria for high-level disinfection (American Association of Tissue Banks, 2016).

Routine sterility tests conducted before tissue packaging revealed a significant tenfold increase in contamination among tissues treated with BASE.128 compared to those treated with the TB cocktail. Notably, there were no other changes to the process. The most prevalent contaminant was *M. simiae*, an NTM commonly found in hospital environments, and a potential human pathogen (Jabbour et al. 2020). The ratio of *M. simiae*-positive homografts appears artificially inflated due to the small number of overall positive cultures, which limits the statistical power and generalizability of these findings. Nevertheless, it is notable that* M. simiae* contamination was detected only following the transition to BASE.128.

The presence of this bacterium in the CVS tissues could result from the cadaveric donor or a contamination acquired during the dissection process. Ciprofloxacin, which has activity against *M. simiae*, is included in the TB cocktail but absent from BASE.128. This omission may have contributed to the persistence of this contaminant in the BASE.128 group. *Streptococcus oralis* and *Prevotella*, part of the normal human microbiome, were also detected in a small number of BASE.128 treated tissues. These bacteria are associated with cardiac and respiratory infections, posing a specific risk to CVS transplant recipients. Further investigation should be carried out regarding their source in the dissected CVS tissues and how to best address it.

Our findings are consistent with reports from other European TBs. A study by Zahra et al. (2019) comparing processing methods in heart valve TBs across Europe found that contamination-related discard rates following antibiotic-based decontamination at 4 °C ranged from 0 to 25%. Tissues discarded due to microbiological detection at the Sheba Tissue and Cell Bank fell within this range, with disposal of 4 out of 296 tissues treated with the TB cocktail (1.4%) and 9 out of 73 tissues decontaminated using BASE.128 (12.3%). This suggests that, while showing some differences in decontamination efficiency, both TB and BASE.128 solutions are effective for processing CVS allografts. Additionally, a strict policy of 100% inspection, i.e., a sterility test for each tissue processed in the Sheba Tissue and Cell Bank , is critical, as microbiological contaminations have been detected in the final stage of tissue production. A possible direction for future investigation would be the incorporation of additional antibiotics into the BASE.128 cocktail to better address contamination by prevalent species such as *M. simiae* and others.

A key limitation of this study is the absence of comprehensive pre-decontamination culture data for the entire study period. Nonetheless, our validation protocol was developed using the prevalent contaminants identified in musculoskeletal tissues processed in our bank, ensuring that the findings remain clinically relevant.

## Conclusion

Decontamination of tissue grafts is crucial to minimize loss due to microbiological contamination and to protect graft recipients from infection. In the absence of standardized procedures, even CE-marked products require validation in each TB, as they are not inherently equivalent or superior to in-house protocols. In our study, BASE.128 was associated with a significantly higher rate of decontamination failure compared to the in-house TB cocktail, underlining the importance of local validation. Each TB should continually monitor and adjust its decontamination protocol to its individual needs, weighing the potential adverse effects of specific antibiotics against the loss of transplants discarded due to insufficient decontamination.

## Data Availability

The datasets generated during and/or analysed during the current study are available from the corresponding author on reasonable request.
